# An Evaluation of the *Fasciola hepatica* miRnome Predicts a Targeted Regulation of Mammalian Innate Immune Responses

**DOI:** 10.3389/fimmu.2020.608686

**Published:** 2021-01-29

**Authors:** Alison Ricafrente, Hieu Nguyen, Nham Tran, Sheila Donnelly

**Affiliations:** ^1^ Faculty of Science, School of Life Sciences, The University of Technology Sydney, Ultimo, NSW, Australia; ^2^ Faculty of Engineering and Information Technology, School of Biomedical Engineering, The University of Technology Sydney, Ultimo, NSW, Australia

**Keywords:** fasciola, helminth, miRNA, immune modulation, dendritic cell, eosinophil, neutrophil

## Abstract

Understanding mechanisms by which parasitic worms (helminths) control their hosts’ immune responses is critical to the development of effective new disease interventions. *Fasciola hepatica*, a global scourge of humans and their livestock, suppresses host innate immune responses within hours of infection, ensuring that host protective responses are quickly incapacitated. This allows the parasite to freely migrate from the intestine, through the liver to ultimately reside in the bile duct, where the parasite establishes a chronic infection that is largely tolerated by the host. The recent identification of micro(mi)RNA, small RNAs that regulate gene expression, within the extracellular vesicles secreted by helminths suggest that these non-coding RNAs may have a role in the parasite-host interplay. To date, 77 miRNAs have been identified in *F. hepatica* comprising primarily of ancient conserved species of miRNAs. We hypothesized that many of these miRNAs are utilized by the parasite to regulate host immune signaling pathways. To test this theory, we first compiled all of the known published *F. hepatica* miRNAs and critically curated their sequences and annotations. Then with a focus on the miRNAs expressed by the juvenile worms, we predicted gene targets within human innate immune cells. This approach revealed the existence of targets within every immune cell, providing evidence for the universal management of host immunology by this parasite. Notably, there was a high degree of redundancy in the potential for the parasite to regulate the activation of dendritic cells, eosinophils and neutrophils, with multiple miRNAs predicted to act on singular gene targets within these cells. This original exploration of the *Fasciola* miRnome offers the first molecular insight into mechanisms by which *F. hepatica* can regulate the host protective immune response.

## Introduction

Fasciolosis is a major production limiting disease of ruminant livestock globally (1). Infection with *F. hepatica*, the liver fluke parasite, results in substantial delays in animals reaching slaughter weight with increased levels of worm burden in the liver directly correlating with reduced growth rates of animals (2). The impact of infection on the production of meat, wool, and milk is estimated to result in economic losses over US$3.2 billion annually (3). Due to the close proximity of people with their livestock, humans are incidental hosts and fasciolosis is now recognized as an emerging human disease. The World Health Organization has estimated that at least 2.4 million people are infected in more than 70 countries worldwide, with several millions at risk, and have thus classified liver fluke infection as one of the food-borne trematode priority diseases (4). Despite this status, the only option to treat the infection is Triclabendazole and although effective at reducing worm burden, it does not prevent re-infection. Furthermore, over reliance on this single drug and its frequent widespread use has resulted in the emergence of resistant flukes (5). The global scale of *F. hepatica* infection, combined with limited treatment options, raises an urgent need to develop novel control strategies. To achieve this, a deeper understanding of the parasite’s mechanisms of invasion and colonisation are necessary.

## *Fasciola hepatica* Manipulates the Host Immune Response to Support Successful Invasion

All mammalian hosts of *F. hepatica* become infected by ingestion of vegetation that is contaminated with the encysted dormant larvae (metacercariae). In the duodenum, the newly excysted juveniles (NEJ) emerge and penetrate the intestinal epithelium to migrate through the peritoneal cavity to reach the liver. Within the liver these parasites spend many weeks feeding on tissue and blood to mature, after which, they migrate to the bile duct, where they take up residence, often for decades, producing thousands of eggs, which are excreted from their mammalian host to continue their life cycle (6).

In naturally infected animals, there is no evidence of the typical host protective, pro-inflammatory Th1 type, immune response that would be expected in response to infection with a pathogen (7). Instead, the Fasciola-specific immune response is predominantly Th2, which becomes more potent as the parasite migrates through the liver. Once the worm is established in the bile duct and the infection becomes chronic, the parasite-specific immune response switches to a combination of regulatory T cells and anergic effector T cells (8–12). Notably, vaccine trials have shown that when a parasite-specific Th1 response is activated, significant levels of protection against infection are achieved (13, 14). Collectively, these observations suggest that by inhibiting the immediate host protective immune response, the parasite ensures survival of the NEJs, permitting their safe passage from the intestine, across the peritoneal cavity and on to the liver, at which point the host response switches to a Th2 phenotype to mediate tissue repair mechanisms. Indeed, in mice deficient in Th2 immunity, worm burden and size were unaffected, suggesting there was no impact to the maturation of the parasite. However, the infected mice displayed significantly more damage to liver tissue and succumbed to premature deaths (15).

While the host innate immune response is activated by the presence of the parasite, resulting in an influx of dendritic cells (DC), eosinophils, neutrophils, and macrophages to the peritoneal cavity immediately after infection (16, 17), evidence suggests that the anti-pathogenic, pro-inflammatory activities of these immune cells are inhibited by the NEJs. The DCs display low expression of CD80, CD40, MHC class II, and CD86 and high expression of CCR5 (17, 18), a phenotype that is indicative of an immature DC. Functionally, these DCs, are unable to promote the differentiation of Th1 cells, and instead induce the expansion of anergic T cells (17). Similarly, macrophages have a low expression of MHC-II and are impaired in their ability to produce pro-inflammatory mediators such as iNOS and TNF, instead adopting a regulatory profile by secreting IL-10 and TGFβ (17, 19, 20). Although not demonstrated *in vivo*, the exposure of bovine neutrophils to the intra-mammalian life stages of *F. hepatica in vitro*, failed to induce significant production of reactive oxygen species or NETosis, suggesting that the parasite was impairing the antimicrobial activities of neutrophils (21). Likewise, there is no evidence of eosinophil degranulation in the peritoneal cavity, which indicates that these cells are not activated or have an alternative phenotype that is not contributing to parasite killing (16).

Understanding the mechanisms that *F. hepatica* employs to disarm the host’s response during the early stages of infection offers the opportunity to counteract these strategies to target the NEJs, which would prevent penetration of the liver capsule and thus the disease pathology. While research to date has largely focused on the characterisation of immune modulating proteins/glycans secreted by helminths during infection [reviewed in (22, 23)], it has recently become apparent that parasitic worms also actively release micro(mi)RNAs which may have a role in the regulation of host immune cells.

## Helminth-Derived miRNAs Regulate Mammalian Gene Expression to Modulate Host Immune Responses

**Figure 1:**
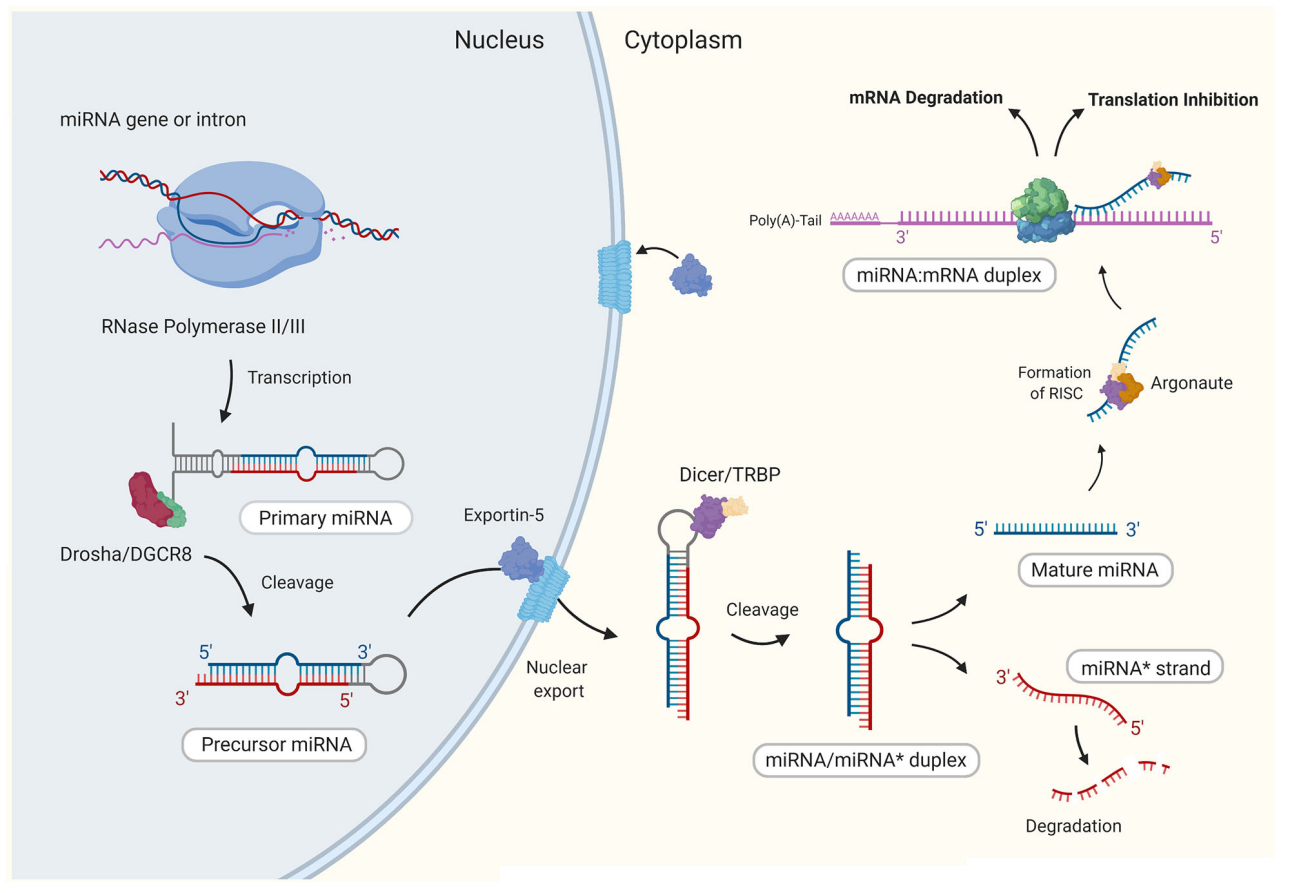
The canonical biogenesis of mammalian mature microRNAs. Generally, through canonical processes miRNAs are first transcribed in the nucleus by RNase polymerase II (Pol-II) to form a stem loop structure that is the primary miRNA (pri-miRNA). Similar to mRNAs, pri-miRNAs present a 5’ 7-methylguanosine (m7G)-cap and polyadenylated 3’ end. The pri-miRNA is then cropped by RNase III Drosha and the double stranded RNA-binding protein DGCR8 (DiGeorge syndrome critical region 8 gene) to form the precursor miRNA (pre-miRNA), which is then exported to the cytoplasm by Exportin-5 (XPO5). In the cytoplasm, the stem loop of the pre-miRNA is cleaved by RNase III Dicer to form a short miRNA duplex (miRNA/miRNA*) comprising of the guide strand and the passenger strand (miRNA*). The miRNA* is degraded while the guide strand is stably loaded onto an Argonaute protein (AGO) which forms the core of the miRNA induced silencing complex (miRISC). Once bound to the miRISC, the mature miRNA finally has the capacity to bind to a target mRNA and form a miRNA/mRNA duplex. Image created by Biorender.

MicroRNAs are small (~22 nucleotides long) non-coding RNA (24), that function to regulate gene expression at the posttranscriptional level through specific binding and subsequent silencing of target messenger RNA (mRNA; **Figure 1**). Target recognition is a highly specific process with complementary binding between the seed region (2–8 nt) at the 5’ end of the miRNA and the 3’ untranslated region (UTR) of the target mRNA (25). This binding activity eventually leads to either inhibition of the initiation step of translation or promotes mRNA decay through the deadenylation of the poly(A)-tail of the mRNA target (25). With many hundreds of human miRNAs identified to date, it is not surprising that miRNAs are recognized to take part in virtually every biological process (26). The first indication that miRNAs were involved in the regulation of immune responses, emerged from a study in 2004, which demonstrated the selective expression of a small number of miRNAs in immune cells (27). Since then, numerous miRNAs have been characterized as having roles in the regulation of both innate and adaptive immune responses, in which they control the development of immune cell progenitors, maintenance and differentiation and mature immune cell function [reviewed in (28)].

Importantly, miRNAs are highly conserved through metazoan evolution and are thus considered to be a vital ancient component of gene regulation (29, 30). Comparative analyses of parasite genomes revealed that a number of helminth miRNAs are widely conserved across diverse organisms and share sequence identity with mammalian species known to have an immune regulatory role (31, 32). These observations led to the suggestion that parasite-derived miRNAs could target mammalian genes within the immune cells of their hosts to modulate immune responses (33). Further support for this hypothesis was provided with the discovery that miRNAs, encapsulated in extracellular vesicles (EVs), secreted by parasitic worms can be delivered to host immune cells (34, 35). While definitive proof for worm-derived miRNAs acting on host cells *in vivo* remains to be obtained, enticing evidence has been provided by *in vitro* studies (34, 36). Of particular relevance to *F. hepatica*, it has been recently reported that EVs, derived from the closely related trematode worm *Schistosoma japonicum*, were internalized by murine macrophages resulting in the release of parasite-derived miRNAs intracellularly. One of these schistosome miRNAs, *sja-miR-125b*, incorporated into the host AGO protein resulting in the regulation of the host Toll Like Receptor (TLR) signaling pathway, which consequently modulated the production of cytokines by the macrophages (36). Despite this growing evidence for a functional role for helminth miRNAs in the host-parasite relationship, proper characterisation of the complete miRNomes of these pathogens is lacking and very little exploration of their putative role in host immune modulation has been performed.

## An Evaluation of the *Fasciola hepatica* miRNome Identified to Date

Currently, the identification of helminth microRNAs is performed from sequencing reads by applying various algorithms based on sequence structure, evolutionary conservation, thermodynamic stability, and machine learning. Consequently, the output from every discovery pipeline is highly dependent on specific input requirements critical to producing reliable miRNA candidates.

The characterization of *F. hepatica* miRNAs has been reported across three primary discovery projects and was achieved using three distinct pipelines mandated by the availability of the *F. hepatica* genome, the sequencing input, and the use of different analytical tools. Initial explorations by Xu et al. (37), employed strategies that compensated for the absence of an assembled *F. hepatica* genome. The availability of a reference genome is required for predicting the candidate pre-miRNAs (characterized by complementary sequences separated by a hairpin loop). These pre-miRNAs give rise to the mature miRNAs that partake in gene regulation. Therefore, without a reference genome, the ability to predict miRNAs from sequencing data is diminished. To fill this gap, Xu et al., utilized the genome of *S. japonicum* in combination with the Short Oligonucleotide Alignment Program (SOAP) to map putative miRNAs within the RNA sequencing data obtained from adult *F. hepatica*. This approach produced the initial repertoire of 16 F*. hepatica* miRNAs. Matching these miRNA candidates with the known miRNAs of *S. japonicum* from miRBase (version 16.0) revealed that eight were conserved miRNAs between the trematode species (**Tables 1** and **2**), which suggested that the remaining eight miRNAs were unique to *F. hepatica*.

**Table 1:** Comparison of miRBase *Fasciola hepatica* mature miRNAs to published sequences.

miR BasemiRNA	Mature miRNASequence on miRBase	Other Proposed Annotation	Other Proposed Mature miRNA Sequence	Study					Sample
				Xu	Fontenla	Fromm 2015	Fromm 2017	Ovchinnikov	
fhe-bantam	TGAGATCGCGATTAAAGCTGGT	Fhe-Bantam		+	+	+	+	+	NEJ, Ad, EV
fhe-miR-2b	GTATCACAGCCCTGCTTGGGACA	miR-2b-3p Fhe-Mir-2-P2b Fhe-Mir-2-P2c	_TATCACAGCCCTGCTTGGGACAC _TATCACAGCCCTGCTTGGGACACA	+	+	+	+	+	NEJ, Ad, EV
fhe-miR-2e	GTATCACAGTCCAAGCTTTGG	miR-2e-3p fhe-mir-2c-A Fhe-Mir-2-P3b	_TATCACAGTCCAAGCTTTGG _TATCACAGTCCAAGCTTTGGT _TATCACAGTCCAAGCTTTGGTAAA	+	+	+	+	+	NEJ, Ad, EV
fhe-miR-10	AACCCTGTAGACCCGAGTTTGCA	miR-10-5p Fhe-Mir-10-P1	AACCCTGTAGACCCGAGTTTG: AACCCTGTAGACCCGAGTTTGC_	+	+	+	+	+	NEJ, Ad, EV
fhe-miR-71a	TGAAAGACGATGGTAGTGAGATG	Fhe-Mir-71-P1b Fhe-Mir-71-P1b	TGAAAGACGATGGTAGTGAGAT_	+	+	+	+	+	NEJ, Ad, EV
fhe-miR-71b	TGAAAGACTTGAGTAGTGAG	Fhe-Mir-71-P1a	TGAAAGACTTGAGTAGTGAGACG	+	+	+	+	+	NEJ, Ad, EV
fhe-miR-124	TTAAGGCACGCGGTGAATGTCA	Fhe-Mir-124	_TAAGGCACGCGGTGAATGTCA	+	+	+	+	+	NEJ, Ad, EV
fhe-miR-2a	GTCACAGCCAGAATTGATGAACG	Fhe-Mir-2-P1b	_TCACAGCCAGAATTGATGAACG	–	+	+	+	+	NEJ, Ad, EV
fhe-miR-2c	ATATCACAGCCGTGCTTAAGGGCT	Fhe-Mir-2-P3a	_TATCACAGCCGTGCTTAAGGGCTT	–	+	+	+	+	NEJ, Ad, EV
fhe-miR-2d	GTATCACAGTCCTGCTTAGGTG	Fhe-Mir-2-P2a	_TATCACAGTCCTGCTTAGGTGACGA	–	+	+	+	+	NEJ, Ad, EV
fhe-miR-2f	GTCACAGCCAATATTGATGCC	Fhe-Mir-2-P1a	_TCACAGCCAATATTGATGCCTG	–	+	+	+	+	NEJ, Ad, EV
fhe-miR-7	TGGAAGACTGGTGATATGTTGTT	Fhe-Mir-7-P1		–	+	+	+	+	NEJ, Ad, EV
fhe-miR-8	CTAATACTGTTTGGTAAAGATGCC	Fhe-Mir-8	_TAATACTGTTTGGTAAAGATGCC	–	+	+	+	+	NEJ, Ad, EV
fhe-miR-9	TCTTTGGTTATCAAGCAGTATG	Fhe-Mir-9	TCTTTGGTTATCAAGCAGTATGA	–	+	+	+	+	NEJ, Ad, EV
fhe-miR-31	TGGCAAGATTATGGCGAAGCTGA	Fhe-Mir-31		–	+	+	+	+	NEJ, Ad, EV
fhe-miR-36a	GTCACCGGGTAGACATTCATTCAC	Fhe-Mir-36-P1	_TCACCGGGTAGACATTCATTCAC	–	+	+	+	+	NEJ, Ad, EV
fhe-miR-61	ATGACTAGAAAGTGCACTCACTT	Fhe-Mir-279	_TGACTAGAAAGTGCACTCACTTC	–	+	+	+	+	NEJ, Ad, EV
fhe-miR-87	GGTGAGCAAAGTTTCAGGTGTGA	Fhe-Mir-87	_GTGAGCAAAGTTTCAGGTGTGA	–	+	+	+	+	NEJ, Ad, EV
fhe-mir-96	CTTGGCACTTTGGAATTGTCA	Fhe-Mir-96	CTTGGCACTTTGGAATTGTCAC	–	+	+	+	+	NEJ, Ad, EV
fhe-miR-125a	TCCCTGAGACCCTAGAGTTTC	Fhe-Mir-10-P2b	TCCCTGAGACCCTAGAGTTTCC	–	+	+	+	+	NEJ, Ad, EV
fhe-miR-125b	CCCCTGAGACTGATAATTGCTC	Fhe-Mir-10-P2a Fhe-Mir-10-P2a	CCCCTGAGACTGATAATTGCT_ CCCCTGAGACTGATAATTGCTCC	–	+	+	+	+	NEJ, Ad, EV
fhe-miR-190	AGATATGTTTGGGTTACTTGGTG	Fhe-Mir-190-P1		–	+	+	+	+	NEJ, Ad, EV
fhe-miR-219	TGATTGTCCATTCGCATTTCTTG	Fhe-Mir-219 Fhe-Mir-219	TGATTGTCCATTCGCATTTCTT_	–	+	+	+	+	NEJ, Ad, EV
fhe-miR-277	GTAAATGCATTTTCTGGCCCG	Fhe-Mir-277-P1	_TAAATGCATTTTCTGGCCCGTAA	–	+	+	+	+	NEJ, Ad, EV
fhe-miR-745b	GAAAGCTGCCAAGCGAAGGGC	Fhe-Mir-22-P2 Fhe-Mir-22-P2	:AAGCTGCCAAGCGAAGGGCCAA :AAGCTGCCAAGCGAAGGGCCAAG	–	+	+	+	+	NEJ, Ad, EV
fhe-miR-2162	GTATTATGCAACATTTCACTCT	Fhe-Mir-1993	_TATTATGCAACATTTCACTCT	–	+	+	+	+	NEJ, Ad, EV
fhe-miR-3479	GTATTGCACTTTCCTTCGCCTTA	Fhe-Mir-92-P1	_TATTGCACTTTCCTTCGCCTTA	–	+	+	+	+	NEJ, Ad, EV
fhe-miR-11584	CCATTATATAAGATTGAGGCTCT	Fhe-Mir-NOV-1	_CATTATATAAGATTGAGGCTCT	–	+	+	+	+	NEJ, Ad, EV
fhe-miR-46	ATGTCATGGAGTTGCTCTCTACA	Fhe-Mir-281 Fhe-Mir-281	_TGTCATGGAGTTGCTCTCTACA AGGAGGGCAATTTTATGACTTT	–	+	+	+	+	NEJ, Ad, EV
fhe-miR-307	ATCACAACCTACTTGATTGAGGGG	Fhe-Mir-67 Fhe-Mir-67	_TCACAACCTACTTGATTGAG:_ CCTCAACAAGAAGGCTGTTGGATG	–	+	+	+	+	NEJ, Ad, EV
fhe-miR-745a	ATGCTGCCTTATAAGAGCTGTG	Fhe-Mir-22-P1 Fhe-Mir-22-P1	_TGCTGCCTTATAAGAGCTGTGA TCAGTTCTCATTAGGCATGACATG	–	+	+	+	+	NEJ, Ad, EV
fhe-miR-1	ATGGAATGTGGCGAAGTATGGT	Fhe-Mir-1-P2 Fhe-Mir-1-P2	_TGGAATGTGGCGAAGTATGG_ _TGGAATGTGGCGAAGTATGGTCT	–	+	–	+	+	NEJ, Ad, EV
fhe-miR-36b	ACCACCGGGTAGACATTCATC	Fhe-Mir-36-P3	_CCACCGGGTAGACATTCATCCGC	–	+	–	+	+	NEJ, Ad, EV
fhe-miR-750	ACCAGATCTGACTCTTCCAGCTCT	Fhe-Mir-750 Fhe-Mir-750	_CCAGATCTGACTCTTCCAGCTCT _CCAGATCTGACTCTTCCAGCTCTT	–	+	–	+	+	NEJ, Ad, EV
fhe-miR-11585	GACCGGTTTCGTCGTTCAACAC	Fhe-Mir-NOV-6 Fhe-Mir-NOV-6	_ACCGGTTTCGTCGTTCAACACC CGTTGCACCGTTCGGAATTCGGGCA	–	+	–	+	+	NEJ, Ad, EV
fhe-let-7	GAGAGGTAGTGACTCATATGACT	fhe-let-7c Fhe-Let-7-P2	_AGAGGTAGTGACTCATATGACT	–	+	–	–	+	NEJ, Ad, EV
fhe-miR-11586	TGTAAGACGATCGTAGTTGACG			–	+	–	–	–	NEJ
fhe-miR-11587	ATTCCGGCAGCTTAGTACAGCT			–	+	–	–	–	NEJ

Ad, Adult F.hepatica; EVs, Adult F. hepatica extracellular vesicles; NEJ Newly excysted juveniles; featured in study (+); not featured in study (-). Sequences that are featured in a study with identical annotation but a non-identical sequence are characterised with a yellow box. Gray highlighted nucleotides represent nucleotide variability and/or missing nucleotides when compared to respective F. hepatica miRBase miRNA. F. hepatica miRBase miRNAs as featured on miRBase database (version 22.1) and hyperlinked to respective miRNA profile on mirbase.org. Other proposed annotations represent the most up to date annotation of the respective other proposed mature miRNA sequence-colour coded based on the study, Xu et al., 2012 (37) (pink), Fromm et al., 2015 (38) (blue), Fromm et al., 2017 (33) (orange) and Ovchinnikov et al. 2020 (39) (green). Sequences sorted based on presence throughout the featured studies.

**Table 2:** Published *Fasciola hepatica* mature miRNAs not featured in miRBase database.

Most Recent Published Annotation	Other Published Annotation	Mature miRNA Sequence(s)	Study					Sample
			Xu	Fontenla	Fromm 2015	Fromm 2017	Ovchinnikov	
Fhe-Let-7-P1	let-7 Fhe-Let-7	GGAGGTAGTTCGTTGTGTGG_ GGAGGTAGTTCGTTGTGTGGT	+	–	+	+	+	Ad, EV
Fhe-Mir-1-P1	fhe-mir-1	TGGAATGTTGTGAAGTATGTAC	–	–	+	+	+	Ad, EV
Fhe-miR-2-P4	fhe-mir-2a-B	TATCACAGCCCTGCTTGGAACA: TATCACAGCCCTGCTTGGAACACA	–	–	+	+	+	Ad, EV
Fhe-Mir-7-P2	fhe-mir-7b	TGGAAGACTTGTGATTAAGTTGT_ TGGAAGACTTGTGATTAAGTTGTT	–	–	+	+	+	Ad, EV
Fhe-Mir-10-P3	fhe-mir-10*	CAAGCTCGGGTATACAGGAGCAG	–	–	+	+	+	Ad, EV
Fhe-Mir-12	fhe-mir-12	TGAGTATTTCATCAAGTAGTG_ TGAGTATTTCATCAAGTAGTGA	–	–	+	+	+	Ad, EV
Fhe-Mir-71-P2	fhe-mir-71b	TGAAAGACATGGGTAATGAGGT	–	–	+	+	+	Ad, EV
Fhe-Mir-184-P1 Fhe-Mir-184-P2	fhe-mir-184	TGGACGGAGATTTGTTAAGAGC	–	–	+	+	+	Ad, EV
Fhe-Mir-1175	fhe-mir-1175	TGAGATTCAACTACTTCAGCTG	–	–	+	+	+	Ad, EV
Fhe-Mir-1992	fhe-mir-1992	TCAGCAGTTGCACCATTGACG	–	–	+	+	+	Ad, EV
Fhe-Mir-1989	fhe-mir-1989	TCAGCTGTGTTCATGTCTTCGA	–	–	+	+	+	Ad, EV
Fhe-Mir-2160-P1	fhe-mir-novel-5 Fhe-Mir-2160	TGGCGCTTAGTTATATGTCATCG	–	–	+	+	+	Ad, EV
Fhe-Mir-NOV-2	fhe-mir-novel-6	AGTGGTGATGGTCGAGTGGTTTAG AGTGGTGATGGTCGAGTGGTTTAG_	–	–	+	+	+	Ad, EV
Fhe-Mir-NOV-3	fhe-mir-novel-7	TCAGCACCGGCCGAAACGACAC TCAGCACCGGCCGAAACGACA	–	–	+	+	+	Ad, EV
Fhe-Mir-92-P2	fhe-mir-92b Fhe-Mir-92-P2	GATTGCACTACTCATAGCCTTC AGGCTGTGTGTAGAGCAAGTTG	–	–	+	+	+	Ad, EV
Fhe-Mir-210-P2	fhe-novel-3 Fhe-Mir-219-P2	TAGTCACTGGGCTACGAACACG TGTGCGTAGTTTCAGTGATTAGC	–	–	+	+	+	Ad, EV
Fhe-Mir-NOV-4	fhe-mir-novel-8	ACCCTCATTTAGATCGAAGGT	–	–	+	+	–	Ad, EV
Fhe-Mir-NOV-5	fhe-mir-novel-10	AGACACTCAGAGGACGATCAGT	–	–	+	+	–	Ad, EV
Fhe-Mir-36-P2		TCACCGGGTGTTTTTCACCCTC GGGTGGATACAGTCGGTTATG	–	–	–	+	+	Ad, EV
Fhe-Mir-190-P2		TGATATGTATGGTTTTCGGTTG	–	–	–	+	+	Ad, EV
Fhe-Mir-277-P2		AAAATGCATCATCTACCCGAGA	–	–	–	+	+	Ad, EV
Fhe-Mir-210-P1		TTGTGCGTCGTTTCAGTGACCGAA	–	–	–	+	+	Ad, EV
fhe-let-7-5p		TGAGGTAGTAGGTTGTATAGT	–	+	–	–	–	NEJ
miR-2e-5p		TACCAACTTAGACTGCGTTAT	–	+	–	–	–	NEJ
miR-61-5p		TGTGAGTCTCTTTCTTGTCCATG	–	+	–	–	–	NEJ
miR-190-3p		CCAGTGACCAAACATATTCTC	–	+	–	–	–	NEJ
miR-10-3p		AAATTCGAGTCTACAAGGAAC	–	+	–	–	–	NEJ
miR-205		CGAGGACGTTCAATGGGTTCT	–	+	–	–	–	NEJ
miR-562		TCTAGTCCGACTTTGTGAGGA	–	+	–	–	–	NEJ
miR-598		CGCTGTACGATGATGATGATTT	–	+	–	–	–	NEJ
miR-920		AGGTGTAGAAGTGGTAACACT	–	+	–	–	–	NEJ
miR-1985		TAAAGTGACTGTTAGAATGGT	–	+	–	–	–	NEJ
miR-2478		TCGTATCCCACCTCTGACACCA	–	+	–	–	–	NEJ
miR-3487		TCCCCGTAATCGAACTGTTGT	–	+	–	–	–	NEJ
fhe-miR-3064		CTGGCTGTTGCGGTTAAACC	–	+	–	–	–	NEJ
fhe-novel-5		TAGAGTACCTGTAGATTTAG	–	+	–	–	–	NEJ
fhe-mir-novel-1		GTGGCCTCGTAGCTCAGCTGGTAG	–	–	+	–	–	Ad, EV
fhe-mir-novel-2		TTTGCATATCTAAGTCGGACA	–	–	+	–	–	Ad, EV
fhe-mir-novel-9		GTCAGCGAAGACGTCGGGAA	–	–	+	–	–	Ad, EV
fhe-mir-novel-11		TCCGAAAACGCGATGGAACCT	–	–	+	–	–	Ad, EV
fhe-mir-novel-12		ATGAGACGGTGAGTGATGAATT	–	–	+	–	–	Ad, EV
fhe-mir-novel-13		GACCGGTGGTGGTCGAGTGGGTT	–	–	+	–	–	Ad, EV
Fhe-Mir-36-P2		TCACCGGGTGTTTTTCACCCTC	–	–	–	+	–	Ad, EV
Fhe-Let-7-P3		GAGGTAGTGAGTTGTATGTCTG	–	–	–	–	+	Ad, EV
Fhe-Mir-36-P2		GGGTGGATACAGTCGGTTATG	–	–	–	–	+	Ad, EV
Fhe-Mir-133		TTGGTCCCTATCAACCAGCTAT	–	–	–	–	+	Ad, EV
Fhe-Mir-278		TCGGTGGGAGTATCATTCGTGC	–	–	–	–	+	Ad, EV
Fhe-Mir-2160-P2		AGGCGCTTTGATTGTCCACACTGA	–	–	–	–	+	Ad, EV

Ad; adult fluke, EV; Adult fluke extracellular vesicles NE; Newly excysted juveniles; featured in study (+); not featured in study (-). Annotation of published miRNA as most up to date of that sequence across studies. Other published annotation represents studies by Xu et al. 2012 (37) (pink), Fromm et al. 2015 (39) (blue), Fromm et al. 2017 (33) (orange), and Ovchinnikov et al. 2020 (39) (green). Sequences that are featured in a study with identical annotation but a non-identical sequence are characterised with a yellow box. Sequences sorted based on presence throughout the featured studies.

The subsequent study by Fromm et al. (38), also worked with the lack of an available *F. hepatica* genome, and instead utilized the miRCandRef tool to develop assembled contigs from *F. hepatica* genomic data (as part of the 50 Helminth Genomes Initiative) to use as the reference genome. This study used a modified version of the miRDeep2 algorithm to permit a higher sensitivity for predicting miRNA loci within the assembled contigs. Using this approach, the sequencing data from the initial study by Xu et al. was re-analyzed in addition to the sequenced miRNA content of extracellular vesicles (EVs) isolated from adult liver fluke. This analysis produced an expanded list of 55 miRNAs, all of which were found in both the adult fluke and the EVs. This list included the eight conserved miRNAs identified by the earlier study (**Table 1** and **2**). However, the eight novel miRNAs proposed in that study were not found by Fromm et al., suggesting that in fact, they may not be *bone fide* miRNAs and thus we propose should be removed from the listed miRNAs for *F. hepatica*.

The most recent discovery project by Fontenla et al. (40), focused on the miRNA content of newly excysted juveniles (NEJ) 6 h post-excystment as opposed to the adult life stage utilized in the previous two studies. Initially, the conservation of *F. hepatica* miRNAs across mammalian and platyhelminth species was determined *via* a series of filtering processes which compared the NEJ sequencing data against published sequences and databases of miRNA and non-coding RNA such as miRBase (version 20.0), Rfam (41) and the functional RNA database fRNAdb (www.ncrna.org/frnadb).

This identified a total of 46 miRNAs. However, although six of these were classified as miRNAs due to high similarity (>85%) with other helminth miRNAs reported in literature, as they were not found within any of the databases the authors removed these from their final list of proposed miRNAs, thus producing a total of 40 (**Tables 1** and **2**). Of these, 34 shared sequence identity to the miRNAs discovered in the previous analyses of the adult miRNA (37, 38), which suggests that the other 6 miRNAs may be specific to the NEJs. Only three of the eight putative novel miRNAs identified by Xu et al. were identified within this sequencing data but were found to correspond to repetitive sequences within the Fasciola genome, confirming the earlier proposition that they are not genuine miRNAs. To differentiate the Fasciola specific miRNAs from conserved sequences within the panel of 40, a genome assembly was generated using genomic sequence reads downloaded from the Welcome Trust Sanger Centre and used as the reference genome for analysis by miRDeep2. Novel mature miRNA candidates were then aligned to *F. hepatica* genomes PRJEB6687 (42) and PRJNA179522 (43) to confirm the presence and location of mature miRNAs. This analysis resulted in the identification of five *F. hepatica* specific miRNAs, of which four are now officially annotated as fhe-miR-11584 to 11587 on miRBase.

Combined, these studies resulted in the identification of 72 miRNAs (excluding the eight proposed novel miRNAs by Xu et al). Of these, 38 are currently featured in the miRBase database (F_hepaticav1; **Table 1**). Of the remaining 34 miRNAs (**Table 2**), 13 were characterized as novel to *F. hepatica* (i.e. unidentified within the miRNA profile of any other species on miRBase). Although the other 21 sequences have been annotated, within their respective publications, based on their similarity with known miRNAs, they are not listed on the miRBase database, suggesting they were not deemed as authentic miRNAs. This is most likely due to expression and biogenesis criteria for miRNA identification. It is possible that miRNAs identified in the adult flukes were not accepted due to the genome assembly that was used in those discovery pipelines. Although several miRNAs in the NEJ study were localized within the Fasciola genome according to the 2015 genome, these miRNAs may not have satisfied the specific criteria for precursor hairpin structures employed by miRBase (44).

However, these should not be discounted as likely miRNAs, as it has more recently been proposed that the hairpin structures of miRNAs may be more variable than originally proposed (45). This suggestion has influenced the development of a new set of criteria for the annotation of metazoan miRNAs, which is employed by the curated miRNA gene database MirGeneDB. Utilising these criteria to re-evaluate the *F. hepatica* small RNA sequencing data from all three of the discovery projects, Fromm et al. (33) determined that the annotated miRNAs, not present in miRBase were genuine miRNAs (**Table 2**) (33). However, seven of the putative novel miRNAs from the Fromm et al., 2015 study (**Table 2**) and 3 novel miRNAs proposed within the NEJ study were no longer considered as *bona fide* miRNAs (**Table 1** and **2**). This outcome highlights the impact of using different criteria for the assessment of miRNAs, as two of these NEJ miRNAs are currently annotated as genuine miRNAs in miRBase as fhe-miR-11586 and fhe-miR11587 (**Table 1**). In addition to the assessment of previously identified miRNAs, this study also discovered eight new conserved miRNAs within the adult parasites (Fhe-mir-1-P2, Fhe-mir-36-P2, Fhe-mir-36-P3, Fhe-miR-190-P2, Fhe-mir-210-P1, Fhe-mir-210-P2, Fhe-mir-277-P2, Fhe-mir-750). Of these, three were also present in the NEJs (Fhe-mir-1-P2, Fhe-mir-36-P3, Fhe-mir-750) and are listed in miRBase as fhe-miR-36b and fhe-miR-750, with the sequences for both Fhe-mir-1-P2, Fhe-mir-36-P3 aligning to fhe-miR-36b (**Table 1**).

As well as using different criteria for assessment, MirGeneDB employs an internal annotation, which differs from the nomenclature utilized by MirBase. Whereas, the miRBase system assigns the next number in succession (i.e. miR-10 was reported after miR-9 etc.) to new sequences, with paralogs indicated by a letter (if there is a difference of a single nucleotide) or a number (if the mature sequences are identical), the MirGeneDB nomenclature was developed to capture the phylogenetic relationship between miRNAs, where genes of common descent are assigned the same miRNA family name (46). Using this system resulted in the re-classification of Mir-novel-5 (33) as a member of the MIR-2160 family (**Table 2**). Similarly, fhe-miR125 has been assigned to the eumetazoan MIR-10 gene family resulting in the nomenclature Fhe-MiR-10, with the paralogs identified as Fhe-Mir-10-P2a and Fhe-Mir-10-P2b (**Table 1**). Likewise, fhe-miR-745a has been named Fhe-Mir-22-P1.

Most recently, the small RNA sequencing data sets from the adult parasites (37) and their EVs (38) were re-evaluated again using an improved version of MirMiner (39). This study reported the discovery of four conserved miRNAs (Fhe-Let-7-P3, Fhe-Mir-133, Fhe-Mir-278 and Fhe-Mir-2160-P2) and four Fasciola-specific miRNAs. Of the parasite-specific miRNAs, the sequences for Fhe-Mir-NOV-1 and Fhe-Mir-NOV-6 were near identical to miRNAs within the NEJ miRBase dataset, listed as fhe-miR-11584 and fhe-miR-11585 respectively (**Table 1**). Although the other two (Fhe-Mir-NOV-2 and Fhe-Mir-NOV-3) may be classified as new sequences, they each differ in only one nucleotide from the previously identified adult parasite miRNAs fhe-mir-novel-6 and fhe-mir-novel-7 respectively (**Table 2**).

Compiling the findings from all of these studies suggests that in addition to the 38 miRNAs listed on miRBase, *F. hepatica* expresses an additional 39 miRNAs (excluding the seven sequences deemed not to be genuine according to the MirGeneDB criteria for miRNA annotation (33), as described above. Of the 77 miRNAs, 36 were identified in both NEJ and adult parasite, 15 were specific to NEJ and 26 were specific to adult parasites (**Tables 1** and **2**).

Despite the similarities in the profiles of miRNAs identified across all studies, it is important to note the number of variations in the recorded sequences for many of the mature miRNAs (**Table 1**). Differences in nucleotides are particularly evident towards the 5’ or 3’ end for 26 of the common mature miRNAs. Studies of mammalian miRNAs have indicated that variations in the 3’- and/or 5’-end(s) of canonical miRNA-sequence represent IsomiRs (sequence variants) created either due to imprecise cleavage of miRNA sequence by drosha or dicer enzymes or through the addition of nucleotides at 3’ end during miRNA-biogenesis (47). Whether the variations within the Fasciola miRNA sequences represent IsomiRs that correlate with different life stages is an issue that will only be resolved with continued analysis of the fluke’s miRNA. Nonetheless, as these changes do not alter the 2–8 nt seed region of these miRNAs, they are not likely to have a significant effect on the specificity of gene that they target.

In contrast, the sequences reported for let-7, miR-1, and miR-71b are quite different between the studies, with nucleotide variations evident throughout the entire sequence of the mature miRNA. Further examination of these sequences suggests that the annotations are correct, but the different sequences may in fact reflect distinct members of the miRNA families (**Table 3**). The sequence of miR-1 identified in the NEJs more closely aligns to the *S. mansoni* miR-1a and other species of fluke, whereas the miR-1 in adult fluke is more closely conserved to *S. mansoni* miR-1b and species of tapeworm. Similarly, miR-71b in NEJs is near identical to only other species of trematode, whereas the adult fluke miR-71b closely resembles other parasitic and non-parasitic helminths outside of the trematode class. Likewise, the sequence of the let-7 within the NEJs is conserved with Planaria while the adult fluke let-7 is closer to other parasitic trematodes and mammalian sequences. This is of particular interest as let-7 is the miRNA that regulates the expression of *Lin41* in *C. elegans*, a gene that controls the transition to adulthood (48). This suggests that perhaps the differences in the listed sequences for Fasciola adult and NEJ miRNAs may in fact reflect the evolution of variants of the same miRNAs specific to the different life stages of Fasciola to ensure the regulation of different gene targets as necessary for maturation of the worm and modulation of host responses in different tissue environments.

**Table 3:** Mature miRNA sequence, study and conservation between fhe-let-7, fhe-miR-1 and fhe-miR-71b.

Source	Species	miRNA	Mature sequence
**Fontenla et al**	***Fasciola hepatica***	**fhe-let-7**	**GAGAGGUAGUGACUCAUAUGACU**
miRBase	*Melibe leonina*	mle-let-7-5p	_UGAGGUAGUGACUCAUUUUGUU
miRBase	*Schmidtea mediterranea*	sme-let-7c-5p	_UGAGGUAGUGACUCAAAAGGUU
miRBase	*Schmidtea mediterranea*	sme-let-7d	_AGAGGUAGUGAUUCAAAAAGUU
**Fromm et al**	***Fasciola hepatica***	**fhe-let-7**	**GGAGGUAGUUCGUUGUGUGGU**
miRBase	*Schistosoma japonicum*	sja-let-7	GGAGGUAGUUCGUUGUGUGGU
miRBase	*Schistosoma mansoni*	sma-let-7-5p	GGAGGUAGUUCGUUGUGUGGU
miRBase	*Ovis aries*	oar-let-7b	UGAGGUAGUAGGUUGUGUGGU
miRBase	*Homo sapiens*	hsa-let-7b-5p	UGAGGUAGUAGGUUGUGUGGUU
**Fontenla et al**	***Fasciola hepatica***	**fhe-miR-1**	**AUGGAAUGUGGCGAAGUAUGGU**
miRBase	*Schistosoma japonicum*	sja-miR-1	_UGGAAUGUGGCGAAGUAUGGUC
miRBase	*Gyrodactylus salaris*	gsa-miR-1-3p	_UGGAAUGUGGCGAAGUAUGGUC
miRBase	*Schistosoma mansoni*	sma-miR-1a-5p	_UGGAAUGUGGCGAAGUAUGG_
**Fromm et al**	***Fasciola hepatica***	**fhe-miR-1**	**UGGAAUGUUGUGAAGUAUGUAC**
miRBase	*Schistosoma mansoni*	sma-miR-1b-3p	UGGAAUGUUGUGAAGUAUGUGC
miRBase	*Echinococcus granulosus*	egr-miR-1-5p	UGGAAUGUUGUGAAGUAUGU_
miRBase	*Echinococcus multilocularis*	emu-miR-1-3p	UGGAAUGUUGUGAAGUAUGU_
**Fontenla et al**	***Fasciola hepatica***	**fhe-miR-71b**	**UGAAAGACUUGAGUAGUGAG**
miRBase	*Schistosoma japonicum*	sja-miR-71b-5p	UGAAAGACUUGAGUAGUGAGACG
miRBase	*Schistosoma mansoni*	sma-miR-71b-5p	UGAAAGACUUGAGUAGUGAGACG
**Fromm et al**	***Fasciola hepatica***	**fhe-miR-71b**	**UGAAAGACAUGGGUAAUGAGGU**
miRBase	*Gyrodactylus salaris*	gsa-mir-71a	UGAAAGACAUGGGUAAUGAGU_
miRBase	*Schmidtea mediterranea*	sme-mir-71c	UGAAAGACAUGGGUAGUGAGAU
miRBase	*Haemonchus contortus*	hco-mir-71	UGAAAGACAUGGGUAGUGAGAC
miRBase	*Heligmosomoides polygyrus*	hpo-mir-71	UGAAAGACAUGGGUAGUGAGAC

Nucleotides highlighted in grey represent mismatches when compared to the F. hepatica mature miRNA sequence (shown in bold text).

Of interest, miR-281, miR-279, miR-67, and miR-1993 identified in adult fluke by Fromm et al. (39), are alternatively annotated as miR-46, miR-61, miR-307, and miR-2162 respectively, by Fontenla et al. (40). An analysis of miR-281 and miR-46 showed that the sequences are in fact very similar, but have been classified as two distinct miRNAs in miRBase. In both fluke miRNA studies; this particular sequence was published as miR-46/miR-281. However, due to increased availability of miRNA sequences within databases, it is now evident that miR-46 is generally found within helminths, and while miR-281 can be found in some species of parasitic helminth it is more prominent in other invertebrates (**Table 4**). This was likely a miRbase consideration to finalize the annotation of this sequence to miR-46 in Fasciola. The same scenario can be applied to the miRNAs characterized as miR-279, miR-67, and miR-1993, which are now listed as miR-61, miR-307, and miR-2162 respectively on miRBase.

**Table 4:** Species conservation of miR-46/281.

Species	miRNA	Mature miRNA Sequence	Phylum			
			Platyhelminth	Nematoda	Anthropoda	Mollusc
***Fasciola hepatica***	**fhe-miR-46**	**ATGTCATGGAGTTGCTCTCTACA**	+			
*Schistosoma mansoni*	sma-miR-281-3p	_TGTCATGGAGTTGCTCTCTATA	+			
*Echinococcus granulosus*	egr-miR-281-3p	_TGTCATGGAGTTGCTCTCTATA	+			
*Echinococcus multilocularis*	emu-miR-281-3p	_TGTCATGGAGTTGCTCTCT__	+			
*Ascaris suum*	asu-miR-46-3p	_TGTCATGGAGTTGCTCTCTTCA		+		
*Panagrellus redivivus*	prd-miR-46-3p	_TGTCATGGAGT_GCTCTCTTA_		+		
*Haemonchus contortus*	hco-miR-46	_TGTCATGGAGTCGCTCTCTTCA		+		
*Heligmosomoides polygyrus*	hpo-miR-46-3p	_TGTCATGGAGTCGCTCTCTTCA		+		
*Caenorhabditis elegans*	cel-miR-46-3p	_TGTCATGGAGGCGCTCTCTTCA		+		
*Caenorhabditis briggsae*	cbr-miR-46	_TGTCATGGAGGCGCTCTCTTCA		+		
*Caenorhabditis brenneri*	cbn-miR-46	_TGTCATGGAGGCGCTCTCTTCA		+		
*Caenorhabditis remanei*	crm-miR-46-3p	_TGTCATGGAGTCGCTCTCTTC_		+		
*Pristionchus pacificus*	ppc-miR-46	_TGTCATGGAGTCGCTCTCTTC_		+		
*Tribolium castaneum*	tca-miR-281-3p	_TGTCATGGAGTTGCTCTCTTT_			+	
*Acyrthosiphon pisum*	api-miR-281	_TGTCATGGAGTTGCTCTCTTT_			+	
*Bombyx mori*	bmo-miR-281-3p	ACTGTCATGGAGTTGCTCTCTT__			+	
*Branchiostoma floridae*	bfl-miR-281	_TGTCATGGAGTTGCTCTCTTTT			+	
*Tetranychus urticae*	tur-miR-281-3p	_TGTCATGGAGTTGCTCTCTTTC			+	
*Manduca sexta*	mse-miR-281	CTGTCATGGAGTTGCTCTCTTT_			+	
*Culex quinquefasciatus*	cqu-miR-281-3p	_TGTCATGGAATTGCTCTCTTT_			+	
*Lottia gigantea*	lgi-miR-281-3p	_TGTCATGGAGTTGCTCTCTTTA				+

Nucleotides highlighted in gray represent mismatches when compared to the Fontenla et al/miRbase F. hepatica mature miRNA sequence (shown in bold text).

This compilation and comparative analysis of the Fasciola miRNome has highlighted the impact and need for appropriate annotation of all flatworm miRNAs. The complexity of miRNA biogenesis giving rise to isomiRs will add to these challenges in annotation and subsequent curation. It must be noted that confirmation and authenticity of miRNAs is largely dependent on available sequencing data, and therefore will improve as more sequencing data is generated and made available. There are currently 38 Fasciola miRNAs listed in miRBase, which is presently the only repository of flatworm miRNAs. As more sequences become available, it is likely that Fasciola will feature within other curated databases such as MirGeneDB and miROrtho. However, it is clear from this compilation of studies, that a universal system of naming needs to be accepted by the wider Fasciola research community for the sake of clarity. The exciting outcome of adopting a uniform system of annotation is the release of a fully curated and annotated Fasciola miRnome for all life stages.

## Predicted Gene Targets for *Fasciola hepatica* miRNAs Reveal a High Degree of Redundancy in the Regulation of Immune Cell Activation

Despite the characterization of *F. hepatica* miRNAs, beyond an acknowledgement that some share homology to mammalian miRNAs involved in the regulation of immune responses (33–37, 38, 40), there has been no detailed exploration of the possible mammalian genes that might be targeted by parasite miRNAs as a mechanism for controlling the host immune response. Given that a single miRNA can have multiple targets and that the 3’UTR of the mRNA can include various binding sites for multiple miRNAs, predictive tools provide an in silico method for screening potential targets followed by experimental validation.

As the miRBase *F. hepatica* miRNAs were identified within the NEJ stage, we specifically explored potential mammalian gene targets that were specific to innate immune cells. It is important to acknowledge that highly characterized species such as human, mouse and rat dominate the curated literature that support these predictive tools and databases. Although *F. hepatica* is typically regarded as a parasite of sheep and cattle, due to the lack of information on the biological contribution of genes in these species it is not possible to accurately apply the predictive tools against these hosts. Accordingly, predictions of mammalian gene targets for Fasciola derived miRNAs were performed against a *Homo sapiens* background. Given that the bovine genome is regarded as 80% similar to the human genome (49), and that the profile of immune response to *F. hepatica* is common across all host species (50), it is probable that the predicted gene targets are common to the regulation of immune response during infection for multiple mammalian hosts of Fasciola.

**Figure 2:**
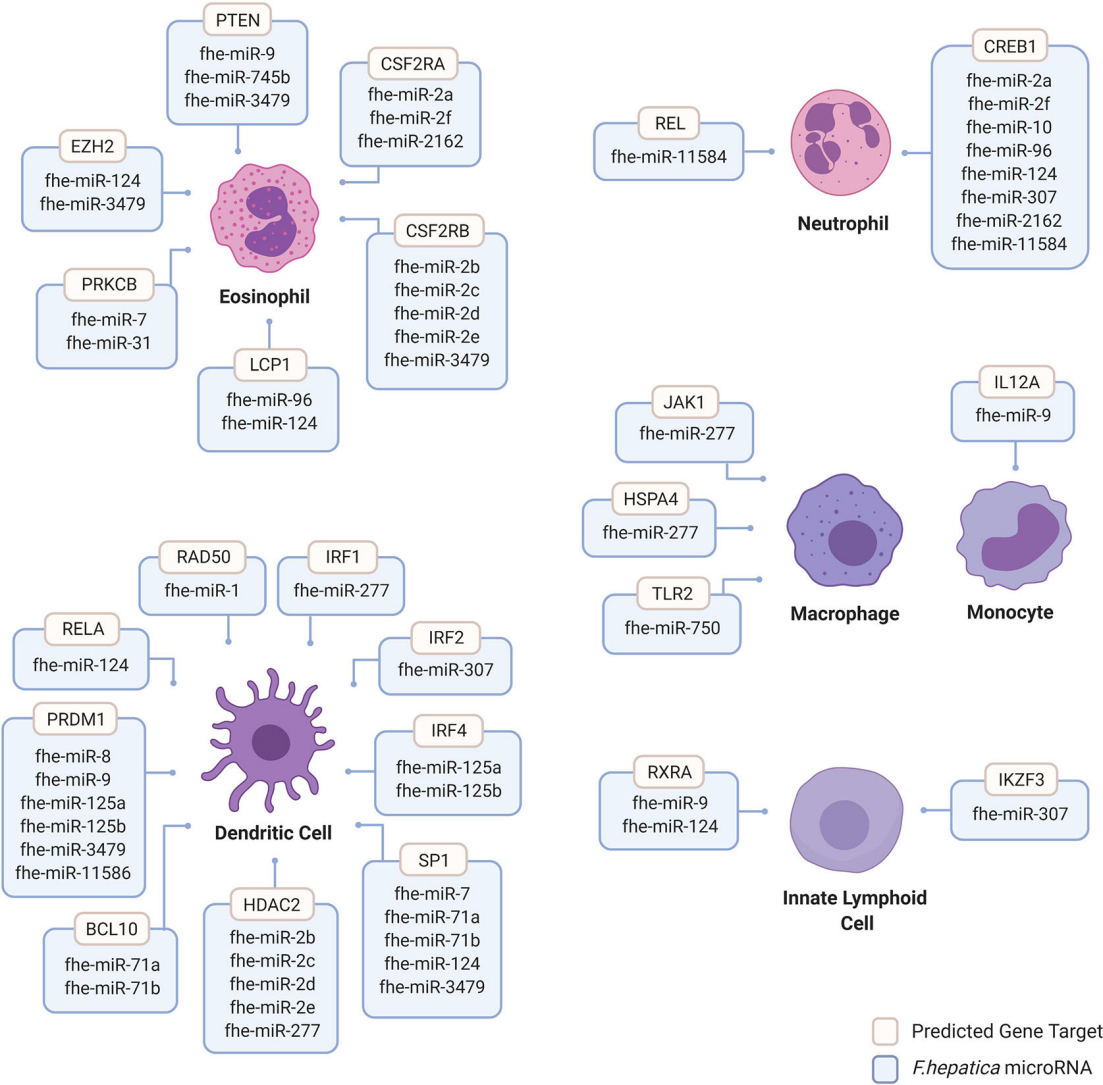
Human innate immune cell genes predicted to be targeted by *Fasciola hepatica* microRNAs. Human genes targeted by *F. hepatica* miRNAs were predicted using the miRNA target prediction tool and database miRDB (mirdb.org). Gene targets are considered with >60 target score produced by miRTarget in miRDB with *Homo sapiens* background using *F. hepatica* miRNA sequences featured in miRbase database version 22 (mirbase.org). Gene targets filtered for innate immune cell background including dendritic cells (DCs), eosinophils, innate lymphoid cells (ILCs), macrophages, monocytes and neutrophils were considered. Dendritic cell targeted genes include B-Cell Lymphoma/Leukemia 10 Signaling Adaptor (BCL10), Histone Deacetylase 2 (HDAC2); Interferon Regulatory Factor (IRF) 1, 2, and 4; Positive Regulatory Domain I-Binding Factor 1 (PRDM1); RAD50 Double Strand Break Repair Protein (RAD50); REL Proto-Oncogene, NF-KB Subunit (RELA) and Sp1 Transcription Factor (SP1). Eosinophil targeted genes include Colony Stimulating Factor 2 Receptor Subunit Alpha (CSFR2A), Colony Stimulating Factor 2 Receptor Subunit Beta (CSFR2B), Enhancer of Zeste 2 Polycomb Repressive Complex 2 Subunit (EZH2), Lymphocyte Cytosolic Protein 1 (LCP1), Protein Kinase C Beta (PRKCB) and Phosphatase and Tensin Homolog (PTEN). Innate lymphoid cell gene targets include Ikaros Family Zinc Finger 3 (IKZF3) and Retinoid X Receptor Alpha (RXRA). Macrophage gene targets include Heat Shock Protein Family A Member 4 (HSPA4), Janus Kinase 1 (JAK1) and Toll Like Receptor 2 (TLR2). Monocyte targeted genes include Interleukin 12A (IL12A). Neutrophil targeted genes include CAMP Responsive Element Binding Protein 1 (CREB1) and REL Proto-Oncogene and NF-KB Subunit (REL). Image created by Biorender.

We analyzed the mature sequences of the 38 miRBase Fasciola miRNAs for custom target prediction using miRDB (mirDB.org.) This database was selected as it utilizes the bioinformatic tool MirTarget, which is a compendium of experimentally validated miRNA targets (51). Genes with a target score of >60 were selected for further analysis using InnateDB (innatedb.com), an integrated analysis platform that has been specifically designed to facilitate systems-level analyses of pathways and genes specific to mammalian innate immune cells (52). Thus, gene targets were further filtered according to their association with immunological responses of innate cells including dendritic cells (DCs), eosinophils, innate lymphoid cells (ILCs), macrophages, monocytes, and neutrophils. Within the miRBase Fasciola miRNome, 26 of the 38 miRNAs were predicted to target genes within all of the innate immune cells examined (**Figure 2**). Of these cells, DCs, eosinophils and neutrophils were the most targeted with 17, 14, and 8 miRNAs, respectively, identified as having gene targets within these cells (**Figure 2**). Furthermore, this mapping revealed a high degree of redundancy as many of the miRNAs were predicted to act on the same targets, suggesting a certain selectivity to the genes that are targeted and thus the modulation to immune response that consequently occurs.

Of the 14 miRNAs acting on eosinophils, 8 were predicted to target colony stimulating factor receptor (CSF2R). This receptor is a heterodimer comprised of an alpha and beta chain. The alpha subunit contains a specific binding site for granulocyte macrophage colony-stimulating factor [GM-CSF; (53)]. The beta chain triggers signal transduction and is also present in the receptor complexes for IL-5 and IL-3 (54–56). Many aspects of eosinophil biology are controlled by GM-CSF and IL-5, acting through the CSF2R complex (57–59). Of relevance to infection with *F. hepatica*, and in particular to the excystment and migration of the NEJs, in the context of intestinal inflammation GM-CSF and IL-5 foster the survival of peripheral eosinophils, but only GM-CSF promotes the activation of effector functions (57). By targeting the expression of both subunits, the Fasciola miRNAs are capable of regulating the recruitment and functionality of eosinophils to reduce parasite killing.

Within DCs, the parasite miRNAs primarily targeted three host genes, Histone deacetylase-2 (HDAC2), PRDM1, and SP1. By regulating their expression, the parasite miRNAs would significantly impact the maturation of DCs, as both HDAC and PRDM1 promote the expression of costimulatory molecules [such as CD40, CD86 and MHC-II (60–62)] and the acquisition of CD1a (63), the hallmark of an IL-12 producing, pro-inflammatory DC. Considering the requirement for DC-derived cytokines and co-stimulatory molecules for efficient T-cell activation, regulating the expression of these genes would also inhibit the development of an effector adaptive immune response, as seen during infection with *F. hepatica*. Indeed knockdown of PRDM1 in DCs resulted in a reduced ability for these cells to induce efficient allogeneic T cell proliferation (62), and inhibition of HDACs in DCs, led to the differentiation of T cells with an anergic phenotype (64).

All eight of the Fasciola miRNAs that were determined to target neutrophils, were predicted to target only a single gene; CREB1. This gene encodes a transcription factor that has a central role in the regulation of the functional response of neutrophils (65–68). Specific mutation of this gene, results in a decreased ability of neutrophils to generate inflammatory chemokines and cytokines (65), which reflects the reduction in neutrophil activity when exposed the parasite.

This analysis strongly supports the hypothesis that parasite-derived miRNAs can regulate host genes, and by doing so can manipulate the functional activity of all immune cells. While, the use of a single miRNA target prediction tool (miRDB) has its limitations, particularly on non-model organisms such as the liver fluke, using it in combination with other databases such as (InnateDB and Reactome) captured preliminary insights to the immunoregulatory functions of Fasciola miRNA. More so, the revelation that multiple parasite miRNAs targeted the same genes within eosinophils, DCs and neutrophils suggest that Fasciola miRNAs have been conserved to regulate specific anti-parasitic immune pathways during an infection. With knowledge of the miRNA sequences, this hypothesis can be experimentally tested.

As this review was being finalized, a similar consideration of the biological role of the 46 of the most abundant miRNAs in adult parasites and EVs was published. Using a combination of TargetScan and PITA to predict gene targets within the genome of cattle and humans, and applying Reactome and KEGG pathway analysis to these targets, identified 44 and 23 genes respectively, that were characterized as having a role in the immune system (39).

## Conclusion

We specifically focused on the innate immune cells as the miRBase list of miRNAs has been validated for the NEJs, a stage of the parasite that is most closely associated with the immediate host immune response. However, based on this analysis the identification of gene targets within every cell suggests that the parasite’s ability to modulate host cell behavior is widespread. Therefore, by characterising the expression of specific miRNAs at each stage of the parasite’s life cycle, this analytical workflow could be extended to other biologically relevant host cells, such as intestinal epithelium, liver cells, B and T lymphocytes, and the bile duct. The outcomes from this analysis would provide a holistic view of the host-parasite crosstalk.

Before undertaking this type of analysis, a definitive characterization of the Fasciola miRNome is required. Compiling the sequencing data and annotations from all five independent studies reporting the identification of the parasite’s miRNAs revealed a surprising level of variation in the sequences and annotation. This outcome illustrates the importance of the research community working together to compare data and to submit these analyses for verification by an independent body. In this manner, the parasite’s miRNome will be correctly catalogued (and reviewed), to be used in future studies as a reference point for comparison and continued expansion. At this stage, the Fasciola research field requires the accurate curation of a completed Fasciola genome and robust guidelines for processing of miRNA sequencing data. If we can generate these tools, variations in sequencing will be reduced and more importantly, it will permit the precise identification of parasite miRNAs within immune cells *in vivo* during an infection. This is an essential piece of defining evidence to fully support the hypothesis that Fasciola miRNAs are manipulating host immune cell function. Furthermore, accurate knowledge of the parasite miRNA sequences would also support the targeted knockdown of specific miRNAs within the NEJs, to determine the relative importance of each in supporting the safe passage of these juvenile parasites to the liver.

One additional consideration is the verification that different isomiRs within the same family of miRNAs may be differentially expressed according to the life stage of the parasite. This possibility was only uncovered as we compared all of the sequences for each annotated miRNA across all of the published studies. It has been shown that the transcriptome of the worm varies greatly between the NEJ and adult stage (69). It is thus, not surprising that the parasite may require different miRNAs to regulate the expression of different parasite genes as it matures. If these isomiRs regulate different targets, this would also represent an adaptation to different immunological environments as the parasite migrates from intestine, through the liver to the bile duct. Further, the fine tuning of single nucleotide in parasitic miRNAs for immune regulation would be a novel area of gene regulation.

In conclusion, the continued characterization and functional analysis of miRNAs in *F. hepatica* will create a new mechanistic framework for the regulation of host immune responses by parasite-secreted miRNAs. This information will also reveal the molecular biological pathways that are unique to parasitism and will be of enormous benefit to the development of novel strategies for infection control.

## Data Availability Statement

Publicly available datasets were analyzed in this study. Details of the original publications and data are provided in the article.

## Author Contributions

SD conceived the idea. SD, AR, and HN searched the literature and drafted the manuscript. NT commented on the structure of manuscript, provided critical intellectual input, and edited the manuscript. All authors contributed to the article and approved the submitted version.

## Funding

AR is a recipient of an Australian Government RTPS Scholarship.

## Conflict of Interest

The authors declare that the research was conducted in the absence of any commercial or financial relationships that could be construed as a potential conflict of interest.

## References

[1] Beesley NJ Caminade C Charlier J Flynn RJ Hodgkinson JE Martinez-Moreno A Fasciola and fasciolosis in ruminants in Europe: Identifying research needs. Transbound Emerg Dis (2018) 65 Suppl 1(Suppl 1):199–216. 10.1111/tbed.12682 28984428 PMC6190748

[2] Mazeri S Rydevik G Handel I Bronsvoort BMD Sargison N Estimation of the impact of Fasciola hepatica infection on time taken for UK beef cattle to reach slaughter weight. Sci Rep (2017) 7(1):7319. 10.1038/s41598-017-07396-1 28779120 PMC5544673

[3] Mehmood K Zhang H Sabir AJ Abbas RZ Ijaz M Durrani AZ A review on epidemiology, global prevalence and economical losses of fasciolosis in ruminants. Microb Pathog (2017) 109:253–62. 10.1016/j.micpath.2017.06.006 28602837

[4] Mas-Coma S Bargues MD Valero MA Human fascioliasis infection sources, their diversity, incidence factors, analytical methods and prevention measures. Parasitology (2018) 145(13):1665–99. 10.1017/S0031182018000914 29991363

[5] Kelley JM Elliott TP Beddoe T Anderson G Skuce P Spithill TW Current Threat of Triclabendazole Resistance in Fasciola hepatica. Trends Parasitol (2016) 32(6):458–69. 10.1016/j.pt.2016.03.002 27049013

[6] Andrews S The life cycle of Fasciola hepatica. In: Fasciolosis, Dalton JP, editor. CABI Publishing (1999). p. 1–29.

[7] Graham-Brown J Hartley C Clough H Kadioglu A Baylis M Williams DJL Dairy Heifers Naturally Exposed to Fasciola hepatica Develop a Type 2 Immune Response and Concomitant Suppression of Leukocyte Proliferation. Infect Immun (2017) 86(1):e00607–17. 10.1128/IAI.00607-17 PMC5736823 28993458

[8] Alvarez Rojas CA Scheerlinck JP Ansell BR Hall RS Gasser RB Jex AR Time-Course Study of the Transcriptome of Peripheral Blood Mononuclear Cells (PBMCs) from Sheep Infected with Fasciola hepatica. PloS One (2016) 11(7):e0159194. 10.1371/journal.pone.0159194 27438474 PMC4954650

[9] Fu Y Chryssafidis AL Browne JA O’Sullivan J McGettigan PA Mulcahy G Transcriptomic Study on Ovine Immune Responses to Fasciola hepatica Infection. PloS Negl Trop Dis (2016) 10(9):e0005015. 10.1371/journal.pntd.0005015 27661612 PMC5035020

[10] Flynn RJ Mulcahy G The roles of IL-10 and TGF-beta in controlling IL-4 and IFN-gamma production during experimental Fasciola hepatica infection. Int J Parasitol (2008) 38(14):1673–80. 10.1016/j.ijpara.2008.05.008 18597757

[11] Mendes EA Mendes TA dos Santos SL Menezes-Souza D Bartholomeu DC Martins IV Expression of IL-4, IL-10 and IFN-γ in the liver tissue of cattle that are naturally infected with Fasciola hepatica. Vet Parasitol (2013) 195(1-2):177–82. 10.1016/j.vetpar.2013.03.035 23648284

[12] Sachdev D Gough KC Flynn RJ The Chronic Stages of Bovine Fasciola hepatica Are Dominated by CD4 T-Cell Exhaustion. Front Immunol (2017) 8:1002. 10.3389/fimmu.2017.01002 28871261 PMC5566560

[13] Golden O Flynn RJ Read C Sekiya M Donnelly SM Stack C Protection of cattle against a natural infection of Fasciola hepatica by vaccination with recombinant cathepsin L1 (rFhCL1). Vaccine (2010) 28(34):5551–7. 10.1016/j.vaccine.2010.06.039 20600503

[14] Molina-Hernández V Mulcahy G Pérez J Martínez-Moreno Á Donnelly S O’Neill SM Fasciola hepatica vaccine: we may not be there yet but we’re on the right road. Vet Parasitol (2015) 208(1-2):101–11. 10.1016/j.vetpar.2015.01.004 PMC4366043 25657086

[15] Stempin CC Motrán CC Aoki MP Falcón CR Cerbán FM Cervi L PD-L2 negatively regulates Th1-mediated immunopathology during Fasciola hepatica infection. Oncotarget (2016) 7(47):77721–31. 10.18632/oncotarget.12790 PMC5363616 27783986

[16] Ruiz-Campillo MT Molina Hernandez V Escamilla A Stevenson M Perez J Martinez-Moreno A Immune signatures of pathogenesis in the peritoneal compartment during early infection of sheep with Fasciola hepatica. Sci Rep (2017) 7(1):2782. 10.1038/s41598-017-03094-0 28584245 PMC5459796

[17] Walsh KP Brady MT Finlay CM Boon L Mills KH Infection with a helminth parasite attenuates autoimmunity through TGF-beta-mediated suppression of Th17 and Th1 responses. J Immunol (2009) 183(3):1577–86. 10.4049/jimmunol.0803803 19587018

[18] Hamilton CM Dowling DJ Loscher CE Morphew RM Brophy PM O’Neill SM The Fasciola hepatica tegumental antigen suppresses dendritic cell maturation and function. Infect Immun (2009) 77(6):2488–98. 10.1128/IAI.00919-08 PMC2687350 19332532

[19] Sulaiman AA Zolnierczyk K Japa O Owen JP Maddison BC Emes RD A Trematode Parasite Derived Growth Factor Binds and Exerts Influences on Host Immune Functions via Host Cytokine Receptor Complexes. PloS Pathog (2016) 12(11):e1005991. 10.1371/journal.ppat.1005991 27806135 PMC5091765

[20] Ruiz-Campillo MT Molina-Hernández V Pérez J Pacheco IL Pérez R Escamilla A Study of peritoneal macrophage immunophenotype in sheep experimentally infected with Fasciola hepatica. Vet Parasitol (2018) 257:34–9. 10.1016/j.vetpar.2018.05.019 29907190

[21] Peixoto R Silva LMR López-Osório S Zhou E Gärtner U Conejeros I Fasciola hepatica induces weak NETosis and low production of intra- and extracellular ROS in exposed bovine polymorphonuclear neutrophils. Dev Comp Immunol (2020) 114:103787. 10.1016/j.dci.2020.103787 32791176

[22] Dalton JP Robinson MW Mulcahy G O’Neill SM Donnelly S Immunomodulatory molecules of Fasciola hepatica: candidates for both vaccine and immunotherapeutic development. Vet Parasitol (2013) 195(3-4):272–85. 10.1016/j.vetpar.2013.04.008 23623183

[23] Maizels RM McSorley HJ Regulation of the host immune system by helminth parasites. J Allergy Clin Immunol (2016) 138(3):666–75. 10.1016/j.jaci.2016.07.007 PMC5010150 27476889

[24] Gantier MP Stunden HJ McCoy CE Behlke MA Wang D Kaparakis-Liaskos M A miR-19 regulon that controls NF-κB signaling. Nucleic Acids Res (2012) 40(16):8048–58. 10.1093/nar/gks521 PMC3439911 22684508

[25] Fabian MR Sundermeier TR Sonenberg N Understanding how miRNAs post-transcriptionally regulate gene expression. Prog Mol Subcell Biol (2010) 50:1–20. 10.1007/978-3-642-03103-8_1 19841878

[26] Vidigal JA Ventura A The biological functions of miRNAs: lessons from in vivo studies. Trends Cell Biol (2015) 25(3):137–47. 10.1016/j.tcb.2014.11.004 PMC4344861 25484347

[27] Chen CZ Li L Lodish HF Bartel DP MicroRNAs modulate hematopoietic lineage differentiation. Science (2004) 303(5654):83–6. 10.1126/science.1091903 14657504

[28] Mehta A Baltimore D MicroRNAs as regulatory elements in immune system logic. Nat Rev Immunol (2016) 16(5):279–94. 10.1038/nri.2016.40 27121651

[29] Wheeler BM Heimberg AM Moy VN Sperling EA Holstein TW Heber S The deep evolution of metazoan microRNAs. Evol Dev (2009) 11(1):50–68. 10.1111/j.1525-142X.2008.00302.x 19196333

[30] Bartel DP Metazoan MicroRNAs. Cell (2018) 173(1):20–51. 10.1016/j.cell.2018.03.006 29570994 PMC6091663

[31] Britton C Winter AD Gillan V Devaney E microRNAs of parasitic helminths - Identification, characterization and potential as drug targets. Int J Parasitol Drugs Drug Resist (2014) 4(2):85–94. 10.1016/j.ijpddr.2014.03.001 25057458 PMC4095049

[32] Fromm B Worren MM Hahn C Hovig E Bachmann L Substantial loss of conserved and gain of novel MicroRNA families in flatworms. Mol Biol Evol (2013) 30(12):2619–28. 10.1093/molbev/mst155 PMC3840308 24025793

[33] Fromm B Ovchinnikov V Høye E Bernal D Hackenberg M Marcilla A On the presence and immunoregulatory functions of extracellular microRNAs in the trematode Fasciola hepatica. Parasite Immunol (2017) 39(2). 10.1111/pim.12399 27809346

[34] Buck AH Coakley G Simbari F McSorley HJ Quintana JF Le Bihan T Exosomes secreted by nematode parasites transfer small RNAs to mammalian cells and modulate innate immunity. Nat Commun (2014) 5:5488. 10.1038/ncomms6488 25421927 PMC4263141

[35] de la Torre-Escudero E Gerlach JQ Bennett APS Cwiklinski K Jewhurst HL Huson KM Surface molecules of extracellular vesicles secreted by the helminth pathogen Fasciola hepatica direct their internalisation by host cells. PloS Negl Trop Dis (2019) 13(1):e0007087. 10.1371/journal.pntd.0007087 30657764 PMC6355031

[36] Liu J Zhu L Wang J Qiu L Chen Y Davis RE Schistosoma japonicum extracellular vesicle miRNA cargo regulates host macrophage functions facilitating parasitism. PloS Pathog (2019) 15(6):e1007817. 10.1371/journal.ppat.1007817 31163079 PMC6548406

[37] Xu MJ Ai L Fu JH Nisbet AJ Liu QY Chen MX Comparative characterization of microRNAs from the liver flukes Fasciola gigantica and F. hepatica. PloS One (2012) 7(12):e53387. 10.1371/journal.pone.0053387 23300925 PMC3534066

[38] Fromm B Trelis M Hackenberg M Cantalapiedra F Bernal D Marcilla A The revised microRNA complement of Fasciola hepatica reveals a plethora of overlooked microRNAs and evidence for enrichment of immuno-regulatory microRNAs in extracellular vesicles. Int J Parasitol (2015) 45(11):697–702. 10.1016/j.ijpara.2015.06.002 26183562

[39] Ovchinnikov VY Kashina EV Mordvinov VA Fromm B EV-transported microRNAs of Schistosoma mansoni and Fasciola hepatica: Potential targets in definitive hosts. Infect Genet Evol (2020) 85:104528. 10.1016/j.meegid.2020.104528 32891875

[40] Fontenla S Dell’Oca N Smircich P Tort JF Siles-Lucas M The miRnome of Fasciola hepatica juveniles endorses the existence of a reduced set of highly divergent micro RNAs in parasitic flatworms. Int J Parasitol (2015) 45(14):901–13. 10.1016/j.ijpara.2015.06.007 26432296

[41] Burge SW Daub J Eberhardt R Tate J Barquist L Nawrocki EP Rfam 11.0: 10 years of RNA families. Nucleic Acids Res (2013) 41(Database issue):D226–32. 10.1093/nar/gks1005 PMC3531072 23125362

[42] Cwiklinski K Dalton JP Dufresne PJ La Course J Williams DJ Hodgkinson J The Fasciola hepatica genome: gene duplication and polymorphism reveals adaptation to the host environment and the capacity for rapid evolution. Genome Biol (2015) 16(1):71. 10.1186/s13059-015-0632-2 25887684 PMC4404566

[43] Martin J Rosa BA Ozersky P Hallsworth-Pepin K Zhang X Bhonagiri-Palsikar V Helminth.net: expansions to Nematode.net and an introduction to Trematode.net. Nucleic Acids Res (2015) 43(Database issue):D698–706. 10.1093/nar/gku1128 PMC4383941 25392426

[44] Ambros V Bartel B Bartel DP Burge CB Carrington JC Chen X A uniform system for microRNA annotation. RNA (2003) 9(3):277–9. 10.1261/rna.2183803 PMC1370393 12592000

[45] Fromm B Billipp T Peck LE Johansen M Tarver JE King BL Uniform System for the Annotation of Vertebrate microRNA Genes and the Evolution of the Human microRNAome. Annu Rev Genet (2015) 49:213–42. 10.1146/annurev-genet-120213-092023 PMC4743252 26473382

[46] Fromm B Domanska D Høye E Ovchinnikov V Kang W Aparicio-Puerta E MirGeneDB 2.0: the metazoan microRNA complement. Nucleic Acids Res (2020) 48(D1):D1172. 10.1093/nar/gkz1016 31642479 PMC7145716

[47] Dhanoa JK Verma R Sethi RS Arora JS Mukhopadhyay CS Biogenesis and biological implications of isomiRs in mammals- a review. ExRNA (2019) 1:3. 10.1186/s41544-018-0003-8

[48] Aeschimann F Neagu A Rausch M Großhans H let-7 coordinates the transition to adulthood through a single primary and four secondary targets. Life Sci Alliance (2019) 2(2):e201900335. 10.26508/lsa.201900335 30910805 PMC6435043

[49] Band MR Larson JH Rebeiz M Green CA Heyen DW Donovan J An ordered comparative map of the cattle and human genomes. Genome Res (2000) 10(9):1359–68. 10.1101/gr.145900 PMC310912 10984454

[50] Cwiklinski K O’Neill SM Donnelly S Dalton JP A prospective view of animal and human Fasciolosis. Parasite Immunol (2016) 38(9):558–68. 10.1111/pim.12343 PMC5053257 27314903

[51] Huang HY Lin YC Li J Huang KY Shrestha S Hong HC miRTarBase 2020: updates to the experimentally validated microRNA-target interaction database. Nucleic Acids Res (2020) 48(D1):D148–54. 10.1093/nar/gkz896 PMC7145596 31647101

[52] Breuer K Foroushani AK Laird MR Chen C Sribnaia A Lo R InnateDB: systems biology of innate immunity and beyond–recent updates and continuing curation. Nucleic Acids Res (2013) 41(Database issue):D1228–33. 10.1093/nar/gks1147 PMC3531080 23180781

[53] McClure BJ Hercus TR Cambareri BA Woodcock JM Bagley CJ Howlett GJ Molecular assembly of the ternary granulocyte-macrophage colony-stimulating factor receptor complex. Blood (2003) 101(4):1308–15. 10.1182/blood-2002-06-1903 12393492

[54] Kouro T Takatsu K IL-5- and eosinophil-mediated inflammation: from discovery to therapy. Int Immunol (2009) 21(12):1303–9. 10.1093/intimm/dxp102 19819937

[55] Desreumaux P Bloget F Seguy D Capron M Cortot A Colombel JF Interleukin 3, granulocyte-macrophage colony-stimulating factor, and interleukin 5 in eosinophilic gastroenteritis. Gastroenterology (1996) 110(3):768–74. 10.1053/gast.1996.v110.pm8608886 8608886

[56] Soman KV Stafford SJ Pazdrak K Wu Z Luo X White WI Activation of Human Peripheral Blood Eosinophils by Cytokines in a Comparative Time-Course Proteomic/Phosphoproteomic Study. J Proteome Res (2017) 16(8):2663–79. 10.1021/acs.jproteome.6b00367 28679203

[57] Griseri T Arnold IC Pearson C Krausgruber T Schiering C Franchini F Granulocyte Macrophage Colony-Stimulating Factor-Activated Eosinophils Promote Interleukin-23 Driven Chronic Colitis. Immunity (2015) 43(1):187–99. 10.1016/j.immuni.2015.07.008 PMC4518500 26200014

[58] Willebrand R Voehringer D IL-33-Induced Cytokine Secretion and Survival of Mouse Eosinophils Is Promoted by Autocrine GM-CSF. PloS One (2016) 11(9):e0163751. 10.1371/journal.pone.0163751 27690378 PMC5045177

[59] Liu LY Wang H Xenakis JJ Spencer LA Notch signaling mediates granulocyte-macrophage colony-stimulating factor priming-induced transendothelial migration of human eosinophils. Allergy (2015) 70(7):805–12. 10.1111/all.12624 PMC9875669 25846339

[60] Nencioni A Beck J Werth D Grünebach F Patrone F Ballestrero A Histone deacetylase inhibitors affect dendritic cell differentiation and immunogenicity. Clin Cancer Res (2007) 13(13):3933–41. 10.1158/1078-0432.CCR-06-2903 17606727

[61] Jiang H Zhang S Song T Guan X Zhang R Chen X Trichostatin a Protects Dendritic Cells Against Oxygen-Glucose Deprivation via the SRSF3/PKM2/Glycolytic Pathway. Front Pharmacol (2018) 9:612. 10.3389/fphar.2018.00612 29942258 PMC6004525

[62] Chan YH Chiang MF Tsai YC Su ST Chen MH Hou MS Absence of the transcriptional repressor Blimp-1 in hematopoietic lineages reveals its role in dendritic cell homeostatic development and function. J Immunol (2009) 183(11):7039–46. 10.4049/jimmunol.0901543 19915049

[63] Cernadas M Lu J Watts G Brenner MB CD1a expression defines an interleukin-12 producing population of human dendritic cells. Clin Exp Immunol (2009) 155(3):523–33. 10.1111/j.1365-2249.2008.03853.x PMC2669529 19220838

[64] Edens RE Dagtas S Gilbert KM Histone deacetylase inhibitors induce antigen specific anergy in lymphocytes: a comparative study. Int Immunopharmacol (2006) 6(11):1673–81. 10.1016/j.intimp.2006.07.001 16979121

[65] Mayer TZ Simard FA Cloutier A Vardhan H Dubois CM McDonald PP The p38-MSK1 signaling cascade influences cytokine production through CREB and C/EBP factors in human neutrophils. J Immunol (2013) 191(8):4299–307. 10.4049/jimmunol.1301117 24038085

[66] Dumitru CA Fechner MK Hoffmann TK Lang S Brandau S A novel p38-MAPK signaling axis modulates neutrophil biology in head and neck cancer. J Leukoc Biol (2012) 91(4):591–8. 10.1189/jlb.0411193 22262799

[67] Sitaraman SV Merlin D Wang L Wong M Gewirtz AT Si-Tahar M Neutrophil-epithelial crosstalk at the intestinal lumenal surface mediated by reciprocal secretion of adenosine and IL-6. J Clin Invest (2001) 107(7):861–9. 10.1172/JCI11783 PMC199578 11285305

[68] Makam M Diaz D Laval J Gernez Y Conrad CK Dunn CE Activation of critical, host-induced, metabolic and stress pathways marks neutrophil entry into cystic fibrosis lungs. Proc Natl Acad Sci U S A (2009) 106(14):5779–83. 10.1073/pnas.0813410106 PMC2667067 19293384

[69] Cwiklinski K Jewhurst H McVeigh P Barbour T Maule AG Tort J Infection by the Helminth Parasite *Fasciola hepatica* Requires Rapid Regulation of Metabolic, Virulence, and Invasive Factors to Adjust to Its Mammalian Host. Mol Cell Proteomics (2018) 17(4):792–809. 10.1074/mcp.RA117.000445 29321187 PMC5880117

